# An Ancient Yeast for Young Geneticists: A Primer on the *Schizosaccharomyces pombe* Model System

**DOI:** 10.1534/genetics.115.181503

**Published:** 2015-10-02

**Authors:** Charles S. Hoffman, Valerie Wood, Peter A. Fantes

**Affiliations:** * Biology Department, Boston College, Chestnut Hill, Massachusetts 02467; † Cambridge Systems Biology Centre and Department of Biochemistry, University of Cambridge, CB2 1GA Cambridge, United Kingdom; ‡ School of Biological Sciences, College of Science and Engineering, University of Edinburgh EH9 3JR Edinburgh, United Kingdom

**Keywords:** education, fission yeast, forward genetics, genetic screen, Model Organism Database, model system, Primer, *Schizosaccharomyces pombe*, *Saccharomyces cerevisiae*

## Abstract

The fission yeast *Schizosaccharomyces pombe* is an important model organism for the study of eukaryotic molecular and cellular biology. Studies of *S. pombe*, together with studies of its distant cousin, *Saccharomyces cerevisiae*, have led to the discovery of genes involved in fundamental mechanisms of transcription, translation, DNA replication, cell cycle control, and signal transduction, to name but a few processes. However, since the divergence of the two species approximately 350 million years ago, *S. pombe* appears to have evolved less rapidly than *S. cerevisiae* so that it retains more characteristics of the common ancient yeast ancestor, causing it to share more features with metazoan cells. This Primer introduces *S. pombe* by describing the yeast itself, providing a brief description of the origins of fission yeast research, and illustrating some genetic and bioinformatics tools used to study protein function in fission yeast. In addition, a section on some key differences between *S. pombe* and *S. cerevisiae* is included for readers with some familiarity with budding yeast research but who may have an interest in developing research projects using *S. pombe*.

## Why Is *Schizosaccharomyces pombe* a Key Model Organism?

FOR students new to research, it may not be obvious why *Schizosaccharomyces pombe* and *Saccharomyces cerevisiae* are such important model organisms. One way to make this point is to describe them as unicellular eukaryotes. As unicellular organisms, they possess many of the same features that in the 1950s and 1960s made the enteric bacterium *Escherichia coli* (along with the bacteriophages that infect it) the premier model organism for molecular biology. Since the entire yeast “organism” is composed of a single cell, one can work with extremely large numbers of individuals to discover rare mutants that eventually identify the genes involved in a biological process of interest. One also can alter the composition of the growth medium and vary the growth conditions (*e.g.*, temperature) to allow for the discovery of genes involved in a wide variety of processes. As eukaryotes, these yeasts can be used to study processes that are conserved from yeast to humans but absent from bacteria, such as organelle biogenesis and cytoskeletal organization, or to study mechanisms such as transcription, translation, and DNA replication, in which the eukaryotic components and processes are significantly different from those of their bacterial counterparts.

Molecular genetics studies in these yeasts benefit from three additional features. First, laboratory strains can be either haploid (with one set of chromosomes) or diploid (with two sets of chromosomes). Haploids are especially useful when screening for mutant alleles that produce a desired phenotype. Since most mutant alleles of a gene will be recessive to the wild-type allele owing to a reduction or loss of function, they would not be detected in a diploid strain but are readily observed in a haploid. Subsequent construction of diploid yeast strains allows one to assess whether a mutant allele is dominant or recessive to a wild-type allele. Yeast diploids are also used to place recessive mutant alleles into complementation groups to determine the number of genes identified in a genetic screen. Second, yeasts can maintain autonomous plasmids that reversibly introduce genetic material to modify the behavior of a strain. This facilitates gene cloning by allowing one to screen plasmids carrying different DNA fragments for those that confer a desired effect on the host strain. Finally, yeasts possess highly active homologous recombination mechanisms. To illustrate this point, 1 cM in genetic distance (a measure of meiotic recombination) is approximately 1 million base pairs (bp) in humans (Kong *et al.* 2004) but only ∼2500 bp in *S. cerevisiae* (Olson *et al.* 1986) and ∼6250 bp in *S. pombe* (Fowler *et al.* 2014). DNA repair systems in budding yeast can promote recombination during vegetative growth (Orr-Weaver *et al.* 1981). Linearized DNA introduced into budding yeast cells is treated by the cells as damaged DNA, leading to recombination with the homologous region of the host chromosome. A similar system exists in fission yeast. The ability to recombine homologous sequences allows researchers to construct strains that carry novel combinations of alleles by (1) the direct introduction of foreign or otherwise modified pieces of DNA to a targeted site in the yeast genome and (2) the introduction of sequences into plasmids by cotransforming a linearized plasmid with a piece of DNA that is flanked by sequences homologous to the site of linearization. In these ways, yeasts are genetically pliable organisms whose biology is well conserved in many respects with that of other eukaryotes.

## What Is *S. pombe*?

*S. pombe*, often known simply as “fission yeast,” is an ascomycete yeast. Ascomycetes are one group of the kingdom Fungi, the other main group being basidiomycetes. Yeasts can belong to either ascomycetes or basidiomycetes but are distinguished from other fungi by their unicellular (as opposed to mycelial) lifestyle and their ability to ferment sugars. The defining characteristic of an ascomycete is that sexual spores are formed within a specialized structure called an *ascus* (Latin for “bag”). In addition to *S. pombe* and *S. cerevisiae*, many filamentous fungi familiar as laboratory organisms such as *Aspergillus* and *Neurospora* are ascomycetes. In contrast, most fungi encountered in the macroscopic world (mushrooms) are basidiomycetes.

On the basis of protein and DNA sequence data, the *Schizosaccharomyces* genus appears to be an ancient “basal” ascomycete (Taphrinomycetes) whose roots go back to the early radiative evolution of ascomycetes and perhaps close to the split between animals and fungi. This makes the evolutionary distance between *S. pombe* and *S. cerevisiae* of the same order as the distance between either of these yeasts and mammals (Sipiczki 2000; Heckman *et al.* 2001; Sipiczki 2004). However, *S. pombe* can be thought of as a more “ancient” yeast than *S. cerevisiae* based on its biological characteristics because it appears to have undergone fewer evolutionary changes since divergence from the common ancestor. For example, *S. cerevisiae* has lost many genes (338) that are conserved between *S. pombe* and mammals (Aravind *et al.* 2000; Wood 2006). Thus, the proteomic content of *S. pombe* is closer to that of the common ancestor. Biological similarities between *S. pombe* and mammals are mentioned elsewhere in this Primer. This is a strong argument for using both yeasts as models. If a process is conserved between the two yeasts, it is likely to be more widely conserved. At the same time, mechanistic differences between the two yeasts underscore the potential for functional diversity among higher eukaryotes.

*S. pombe* is widely distributed around the world and has been isolated from a variety of natural sources. *Pombe* is the Swahili word for “beer” (or at least a beer-like fermented beverage), and *S. pombe* is used for its fermentation. A word of warning: in the authors’ experience, beer produced by *S. cerevisiae* is far more palatable! *S. pombe* also has been isolated from fruits; from kombucha, a tea product produced by mixed fermentation with yeasts (including *S. pombe*) and bacteria; and from molasses, used to produce distilled spirits such as rum and tequila (Gomes *et al.* 2002). Another use of a *Schizosaccharomyces* yeast derives from its ability to utilize malic acid and thereby reduce undesirable acidity in wine (Volschenk *et al.* 2003).

## Origins of *S. pombe* Research

*S. cerevisiae* has been a companion to humans since the invention of bread making and brewing. In contrast, apart from the relatively minor applications mentioned earlier, *S. pombe* has not historically had many practical applications. This difference has influenced the ways in which these two model organisms were used in scientific research. Because of interest in improving brewing and baking methods, there is a long history of studying *S. cerevisiae* physiology that has focused on the regulation of metabolism. Once genes could be cloned, this led to studies on how gene expression is regulated in response to environmental (growth) conditions and specific genetic changes. Later, researchers investigated cell biological aspects such as cell cycle control, the cytoskeleton, mating processes, and so on. The mass of information about the roles and regulation of genes derived from studies on metabolic genes was exploited in early genetic engineering experiments, *e.g.*, in making expression vectors. In contrast, the main focus in *S. pombe* has been on basic interest-driven research. Research started in the 1940s and early 1950s in two main areas: the mating-type system, which led to investigation of the sexual cycle, and the growth and division processes that comprise the cell division cycle.

The founder of *S. pombe* genetics was Urs Leupold, a Swiss student who visited the Carlsberg Laboratory in Copenhagen during the 1940s. He was introduced to the yeast by Øjvind Winge, head of the Carlsberg Laboratory and “father” of *S. cerevisiae* genetics, who suggested the genetic basis of *S. pombe* homothallism as the subject for Leupold’s Ph.D. thesis (Leupold 1993). Unfortunately, the strain Leupold was given proved to be infertile, but he later obtained a high-fertility isolate from Delft, Netherlands. This latter isolate proved to contain a homothallic strain and two heterothallic strains apparently derived from it (see later). These strains, 968 *h*^90^, 972 *h*^−^, and 975 *h*^+^, are the ancestor strains for almost all genetic studies on *S. pombe*. Leupold showed that the differences between the strains were due to alleles of a single genetic locus, now known to be a complex region with silent and expressed genes. Because all three strains derive from a single isolate, the strains used in laboratories around the world are nearly isogenic. This avoids some of the problems experienced in *S. cerevisiae* studies, in which genetic differences between “wild-type strains” such as S288c and W303 (Ralser *et al.* 2012) can cause these strains to respond differently to additional mutations (Elion *et al.* 1991). Leupold and collaborators went on to develop *S. pombe* as a genetically tractable organism by isolating a range of mutants and constructing chromosome maps. His laboratory at the University of Bern, where he became head of the Institute of General Microbiology, remained the main *S. pombe* genetics center for several decades.

At around the same time, Murdoch Mitchison, working in Edinburgh, was interested in how single cells grew in mass between cell divisions. He tried various cell types, from bacteria to sea urchin eggs, and settled on *S. pombe* when he realized that its mode of growth by linear extension and medial division allowed the age of a single cell to be fairly accurately determined by a single cell length measurement (Mitchison 1990). His main interests were in the patterns of increase during the cell cycle of such cellular properties as total cell mass and in the rates of overall protein synthesis of individual proteins and other macromolecules. It was thought that division might be triggered by the accumulation of a molecule to a critical level. Thus, the identification of molecules whose abundance increased during the cell cycle might lead to insight into the control of division (Mitchison 1971). Indeed, such molecules—the mitotic cyclins—were shown some 20 years later to be key regulators of mitosis.

The genetics and cell cycle strands of *S. pombe* research came together in the mid-1970s when Paul Nurse, following the pioneering work of Lee Hartwell on *S. cerevisiae*, undertook the isolation of *S. pombe* cell division cycle (*cdc*) mutants in Mitchison’s laboratory, having first spent time in Leupold’s group, where he learned genetics methods. In the mid-1970s, a series of papers published by Nurse and colleagues described cell cycle mutants and their use in investigating how the cell cycle is controlled (Fantes 1989).

For two decades, the *S. pombe* field was dominated by the laboratories headed by Leupold and Mitchison and later by their scientific progeny, students and postdoctoral fellows who had worked in their laboratories. Meanwhile, there was a steady accumulation of knowledge about *S. pombe* by European researchers such as Herbert Gutz, Henri Heslot, and Nicola Loprieno (Gutz *et al.* 1974). A major step forward was taken by Richard Egel, who isolated and characterized mutants unable to undergo meiosis (Bresch *et al.* 1968; Egel 1973). This represented the first use of a genetics approach to study the *S. pombe* life cycle, and Egel’s mutants formed the basis for many subsequent studies.

**Figure 1:**
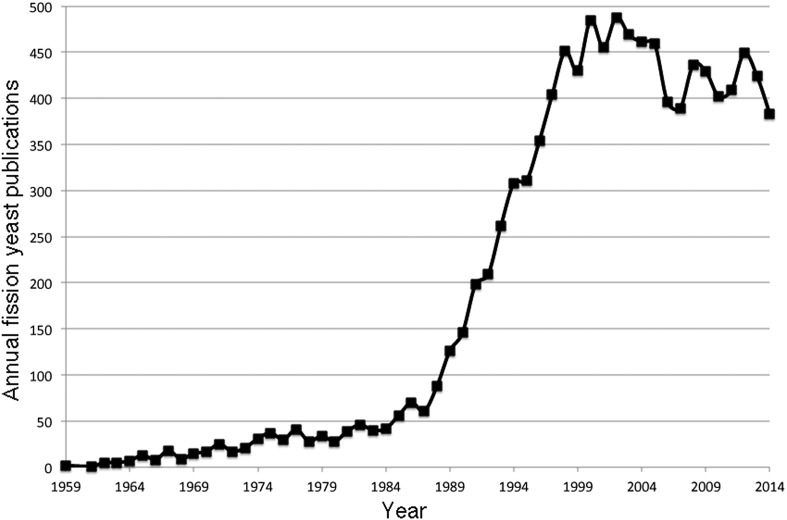
*S.pombe* publication numbers from 1959 to 2014.

During the following decades, *S. pombe* research expanded worldwide, and this led to the First International Fission Yeast Meeting held in Edinburgh in 1999. Since then, *S. pombe* has gone from strength to strength, as shown by the increase in fission yeast publications over the years (Figure 1).

## *S. pombe* Genome Content

In 2002, fission yeast became the sixth eukaryotic model organism to have its genome sequence and annotation published (Wood *et al.* 2002). The genome sequence provided a robust platform for the ongoing evaluation of genome content, both by the annotation of features on the sequence and by attaching functional annotations to those features. Gradually, a more complete picture of *S. pombe* is emerging as new data sets from both small- and large-scale experiments are aggregated to refine the annotated gene structures and assign functional data to them.

### Gene complement

A central goal of biological research is to describe fully the orchestrated collection of functions and processes that combine to produce living cells. One way to assess progress is by maintaining and interrogating an accurate “parts list” of the contributing gene products and their attributes. The *S. pombe* parts list is maintained by the model organism database (MOD) PomBase (http://www.pombase.org), with 5054 protein-coding genes currently reported (compared to 5821 for *S. cerevisiae*). This difference in protein number is partly due to the retention of many paralogous pairs (homologous genes within an organism) in *S. cerevisiae* following what appeared to have been two rounds of whole-genome duplication (WGD) (Wolfe and Shields 1997; Kellis *et al.* 2004). Despite a lower number of protein-coding genes, fission yeast has a large number of proteins (currently 338) that are conserved in vertebrates (source: PomBase Query Builder using curated fission yeast/budding yeast and fission yeast/human orthologs) but absent from budding yeast, while only 179 genes are conserved between budding yeast and vertebrates that are absent from fission yeast (source: SGD Yeastmine using Compara human orthologs and PomBase curated fission yeast/*cerevisiae* orthologs supplemented by manual curation). This is a consequence of the increased lineage-specific gene losses in the budding yeast lineage after fission and budding yeasts diverged from a common ancestor (Aravind *et al.* 2000; Wood 2006).

Of the 5054 known or predicted fission yeast proteins, at this time, 2154 have a published biological role, 2050 have a biological role inferred from an experimentally characterized ortholog (usually from budding yeast), and 850 have no known biological role [inferred from the absence of Gene Ontology (GO) biological process annotation]. A large number of these uncharacterized proteins are conserved outside the *Schizosaccharomyces* genus; 182 are conserved in mammals, almost 40% (68) of which have no budding yeast ortholog. Tantalizingly, a number of these genes are orthologs of human disease genes or members of the Catalogue of Somatic Mutations in Cancer (COSMIC) of genes with mutations in cancer genomes (Forbes *et al.* 2014). For example, SPAC652.01 ortholog BLCAP is associated with bladder cancer, and SPAC6G10.10c ortholog MMTAG2 is associated with multiple myelomas. Characterization of these gene products in fission yeast almost certainly will shed light on the functions of human proteins.

Noncoding RNAs (ncRNAs) include all RNAs other than messenger RNA (mRNA) and are central to a wide range of biological processes, including transcription, translation, gene regulation, and splicing. The ncRNA annotation of the fission yeast genome has increased dramatically in recent years. In addition to the 307 ribosomal RNAs (rRNAs), transfer RNAs (tRNAs), small nuclear RNAs (snRNAs), and small nucleolar RNAs (snoRNAs), there are now 1538 additional ncRNAs annotated in the genome, mainly from RNA sequencing (RNA-Seq) data (Watanabe *et al.* 2002; Wilhelm *et al.* 2008; Rhind *et al.* 2011). Similar extensive intergenic and antisense transcription is detected in budding yeast (Yassour *et al.* 2010; Goodman *et al.* 2013; Pelechano *et al.* 2013). A large number of the ncRNAs identified in fission yeast (694) appear to be antisense to protein-coding genes, and some (*e.g.*, *tos1*, *tos2*, and *tos3*, which are antisense to *rec7*) carry out a regulatory role during sexual differentiation (Molnar *et al.* 2001; Watanabe *et al.* 2002), whereas many others display meiosis-specific expression (Bitton *et al.* 2011). Of the remainder, only a handful, including BORDERLINE/IRC1 (Cam *et al.* 2005; Keller *et al.* 2013), *mrp1* (Paluh and Clayton 1995), *rrk1* (Krupp *et al.* 1986), *sme2* (Watanabe and Yamamoto 1994), and *srp7* (Ribes *et al.* 1988) are characterized, hinting at a massive potential for future expansion of ncRNA research in fission yeast. Most of the annotated ncRNAs are of low abundance in vegetative cells, similar to tightly repressed meiotic genes, but 187 of them are expressed at ∼1–200 copies/cell (Marguerat *et al.* 2012).

### Chromosome organization

The *S. pombe* genome size is ∼13.8 Mb compared to the 12.5-Mb genome of *S. cerevisiae*. Despite similar genome sizes, *S. pombe* has only 3 relatively large chromosomes of 5.7, 4.6, and 3.5 Mb compared to 16 smaller chromosomes in *S. cerevisiae*. Indeed, at 3.5Mb, the smallest *S. pombe* chromosome (chromosome III) is over twice the length of the largest *S. cerevisiae* chromosome (chromosome I), which is 1.5 Mb.

Fission yeast has large modular centromeres, more reminiscent of those of higher organisms than of the 125-bp element sufficient for centromere function in *S. cerevisiae*. Fission yeast centromeres (*cen1*, *cen2*, and *cen3*) are approximately 40, 69, and 110 kb. The centromere structure comprises a nonconserved central core sequence flanked by variable numbers of outer repeats. Many of the proteins that bind to *S. pombe* centromeres are conserved in mammals but absent from budding yeast, including Swi6 and Chp1 and the RNA interference (RNAi) pathway components (Lorentz *et al.* 1994; Ekwall *et al.* 1995; Doe *et al.* 1998; Volpe *et al.* 2002). The conservation of centromere features, including size, structure, and multilayered organization, makes fission yeast a valuable model for the study of eukaryotic chromatin remodeling and centromere function.

Subtelomeric regions in both fission yeast and budding yeast are reversibly transcriptionally silent (Aparicio *et al.* 1991; Nimmo *et al.* 1998; Halme *et al.* 2004) and are enriched for genes encoding membrane transporters, stress-response proteins, and expanded families of species-specific cell surface glycoproteins (Goffeau *et al.* 1996; Robyr *et al.* 2002; Wood *et al.* 2002; Hansen *et al.* 2005). In fission yeast, these regions are very similar to each other (approximately 50–60 kb of the sequence immediately proximal to the telomeric repeats has ∼99% identity among telomeres for most of the region). A similar localization of contingency genes involved in antigenic variation for immune evasion has been observed in parasitic microbes (Barry *et al.* 2003). Subtelomeric regions therefore may provide the ideal genomic location for eukaryotic cells to test and select for novel species-specific genes required for stress and surface variation in response to changing environmental conditions, and the over-representation of environmentally regulated genes in subtelomeres may be applicable to eukaryotes in general.

Finally, the *S. pombe* genome contains 262 sequences comprising partial or complete copies of mobile genetic elements (Bowen *et al.* 2003) derived from LTR retrotransposons. Thirteen full-length active copies of Tf2 exist in the classic laboratory strains, while active copies of Tf1 only occur in wild isolates of *S. pombe* (Levin *et al.* 1990; Levin and Boeke 1992; Hoff *et al.* 1998). Although these are expressed at low levels under standard laboratory conditions, forced induction of retrotransposon transcription leads to integration of new copies into promoters of RNA pol II–transcribed genes (Guo and Levin 2010). Interestingly, Tf1 integration favors stress-response gene promoters, and once integrated, the Tf1 generally increases expression of the adjacent gene (Guo and Levin 2010; Feng *et al.* 2013). Thus, these mobile elements may serve as a source of genetic variability to promote adaptation to new conditions of environmental stress.

## Genetic Nomenclature

All *S. pombe* genes have a systematic identifier, while many, but not all, have community-assigned gene names that bear some similarities to those of *S. cerevisiae* in that they follow the convention of three letters and a number written in italics, such as *wee1*. However, unlike *S. cerevisiae* genes, *S. pombe* genes are always written in lowercase. This contrasts with *S. cerevisiae* nomenclature, which uses capital letters to designate either wild-type or dominant mutant alleles. For *S. pombe* genes, one uses a superscript plus sign to indicate a wild-type allele (*wee1*^+^), a hyphen and allele number to indicate a mutant allele (*wee1-50*), and a Greek delta or a capital *D* to indicate a deletion allele (*wee1*Δ or *ura4-D18*). Alternatively, deletions may be indicated by a pair of colons followed by the selectable marker that was inserted into that locus (*g*ene *o*f *i*nterest 1 deleted with the *ura4*^+^ gene would be *goi1*::*ura4*^+^), while a tagged allele may be indicated by a hyphen (*goi1*-GFP). *S. pombe* proteins are not italicized and are written by a capital letter followed by lowercase letters (Wee1).

## Life Cycle

The life cycle of *S. pombe* consists of asexual (vegetative) and sexual phases, and these have been described many times (Egel 1989; Fantes 1989; Forsburg and Nurse 1991). Without a doubt, *S. pombe*’s greatest claim to fame as a model organism comes in the area of cell cycle control—how progress through the vegetative cycle is regulated by internal and external cues (see the section *Notable Advances from Research on Fission Yeast* for the discoveries that led to Lasker and Nobel Prizes for Paul Nurse). Its sexual cycle also has been the subject of many studies, starting in the 1950s with Leupold’s work on mating types and followed by Egel’s pioneering work (Egel 1973, 1989; Egel and Egel-Mitani 1974; Leupold 1993).

### Mitotic cell cycle

**Figure 2:**
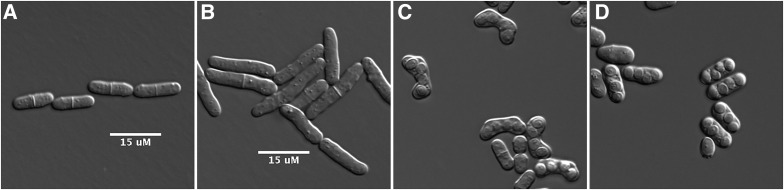
*S. pombe* cells and asci. (A) A haploid cell containing a fission plate. (B) Diploid cells. (C) Zygotic asci formed by the mating of two haploid cells. (D) Azygotic asci formed when diploid cells go through meiosis.

Mitchison’s adoption of *S. pombe* as an ideal organism in which to study the cell cycle (Mitchison 1990) was based on the modes of growth and division of the cells. *S. pombe* cells are cylindrical in shape with roughly hemispherical ends and are about 3.5 µm in diameter during exponential growth on nutrient-rich medium. Newborn cells are about 8 µm long and grow continuously through most of the cell cycle without width change. This means that a good estimate of the age of a cell (time elapsed since birth) can be obtained simply by measuring its length. When cells reach about 15 µm in length, they undergo a closed mitosis (as with most fungi, the nuclear envelope does not break down) with the nucleus close to the center of the cell. Shortly after nuclear division, a transverse septum is laid down medially, which is then cleaved to produce two daughters (Figure 2A and supporting information, File S1). The symmetric mode of division means that the two daughter cells are nearly equal in size, similar to cultured mammalian cells, but quite different from the asymmetric division of budding yeast. The nuclear DNA replicates very soon after mitosis (Nasmyth *et al.* 1979). Thus, the short G_1_ phase takes place between mitosis and cell separation, with two unreplicated nuclei present in the cell until the septum is cleaved. S phase is coincident with the presence of the septum, so newborn daughter cells are already in G_2_, containing fully replicated chromosomes. After cytokinesis, cells remain in a G_2_ phase, which occupies about three-quarters of the cell cycle, leading up to the next mitosis. As such, virtually every haploid cell in a growing population contains two copies of the chromosomal complement. Diploid *S. pombe* cells are noticeably wider (∼4.4 µm) than haploid cells and undergo cytokinesis at approximately 24 µm in length (Figure 2B and File S2), making them almost double the volume of haploid cells.

When subjected to nutrient starvation in the absence of a mating partner, haploid cells exit the cell cycle and enter stationary phase. One unusual feature of *S. pombe* is that while they enter stationary phase from G_1_ on nitrogen starvation, they do so from G_2_ upon glucose starvation (Costello *et al.* 1986). If a mating partner is present, haploid cells shift from vegetative growth to a sexual cycle (described in the next section). Depending on their mating-type constitution (see next section), diploid cells enter either stationary phase or the sexual cycle in response to nutrient starvation.

### Sexual cycle and mating-type system

**Figure 3:**
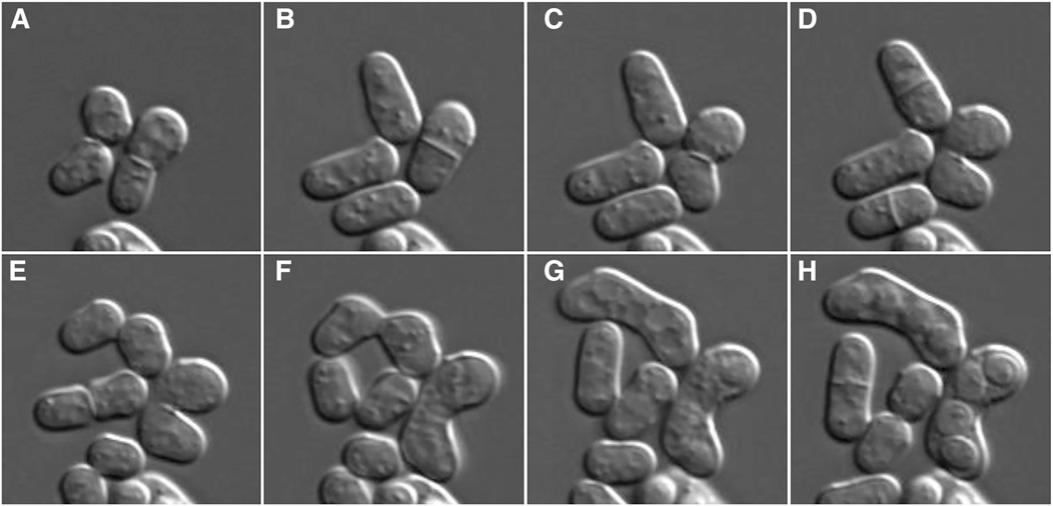
Sexual development in *S. pombe*. Cells of the homothallic *h*^90^ mating type were plated onto a thin EMM agar pad at 30° and photographed at (A) 0 min, (B) 184 min, (C) 204 min, (D) 254 min, (E) 286 min, (F) 334 min, (G) 446 min, and (H) 560 min. Because *h*^90^ cells can undergo mating-type switching, which occurs in only one of the two daughter cells, two pairs of daughter are seen to have mated and formed asci.

In addition to vegetative growth, *S. pombe* has a sexual cycle. Two haploid cells of appropriate mating types (see later) respond to nutrient starvation by mating (conjugation) (Egel and Egel-Mitani 1974; Egel 1989). This involves adhesion of the cells followed by digestion of the walls separating them to form a single fusion cell containing the two parent nuclei (Figure 3 and File S3). The nuclei then fuse to form a zygote, a single cell containing a single diploid nucleus. Normally, the nucleus enters the meiotic pathway soon after zygote formation, and four haploid nuclei are generated. Subsequently, a spore wall forms around each nucleus to produce an ascus consisting of four spores within the shell of the zygote (Figure 2C and Figure 3H). These zygotic asci typically have a bent shape that reflects the angle between the two cells that fused to generate the zygote (Figure 2C). In contrast, azygotic asci derived from vegetative diploid cells are short and linear (Figure 2D). Whether zygotic or azygotic, the ascus wall lyses on exposure to growth medium, and the spores germinate and develop into vegetative cells that return to mitotic growth.

**Figure 4:**
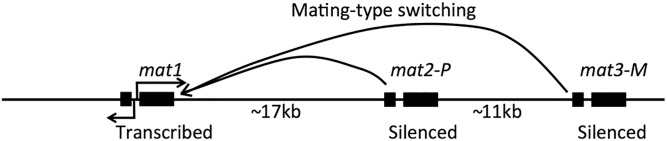
Schematic of the *S. pombe* mating-type locus.

Truly wild-type *S. pombe* strains are of the homothallic mating type referred to as *h*^90^, in that they possess three copies of information at the mating-type (*mat*) locus, one of which is expressed and two of which are transcriptionally silenced (Figure 4). *S. pombe h*^90^ strains can transfer genetic information from either silent locus to the expressed locus. This produces a mixture of cells expressing one or the other mating type within a clone to allow mating to take place. This may be beneficial to surviving starvation conditions in the wild by allowing for the creation of spores. *S. pombe* cells also can be heterothallic such that they require a mating partner of the opposite mating type (Leupold 1950).

### Diploids

After cells mate, the diploid zygote generally undergoes meiosis and sporulation immediately to produce a zygotic ascus. This depends on the mating-type composition of the diploid: *h*^−^ and *h*^+^ haploid parents will produce an *h*^−^/*h*^+^ diploid that is meiosis (and sporulation) competent, whereas a diploid that is homozygous for either the *h*^+^ or *h*^−^ mating type is not able to enter meiosis. Mating within an *h*^90^ culture or of *h*^90^ cells with *h*^−^ or *h*^+^ cells will also produce a meiosis-competent diploid. The normally transient diploid state of a cell can be “rescued” by transfer to nutrient-rich growth medium. A fraction of such diploid cells will propagate vegetatively as diploids, though rearrangements at the *mat* locus and/or spontaneous meiosis and sporulation are frequent events. The practice of making and using diploid *S. pombe* strains is discussed further later.

## Vive la Difference

It has not escaped our notice that substantially many more people have worked with *S. cerevisiae* than with *S. pombe*. Thus, we take this opportunity to point out some biological differences between the two yeasts that one must take into account when transitioning from the lower yeast side to this higher yeast. This Primer is not an attempt to describe all known methods for *S. pombe* molecular genetics research. We therefore point the reader to several invaluable sources of information. These include two Cold Spring Harbor Laboratory manuals, *Experiments with Fission Yeast* (Alfa *et al.* 1993) and *Fission Yeast: A Laboratory Manual* (Hagan *et al.* 2016); the books *Molecular Biology of the Fission Yeast* (Nasim *et al.* 1989) and *The Molecular Biology of Schizosaccharomyces pombe* (Egel 2004); volume 33, issue 3 of the journal *Methods* (edited by Kathleen Gould); and individual methods chapters written by Moreno, Klar, and Nurse (Moreno *et al.* 1991) and by Forsburg and Rhind (Forsburg and Rhind 2006). There is also a wealth of information regarding *S. pombe* on Susan Forsburg’s PombeNet website (http://www-b*cf*.usc.edu/∼forsburg/). If all else fails, or if you just want to get to know us better, you can always e-mail questions to the *S. pombe* mail list hosted by the European Bioinformatics Institute at pombelist@ebi.ac.uk.

### Growth media

It is common for people who have experience with *S. cerevisiae* to simply use the same growth medium when starting to work with *S. pombe*. We strongly recommend against the use of YPD (the rich medium used for *S. cerevisiae*) for *S. pombe*. Rich medium for *S. pombe* (YES) is made with only yeast extract, glucose, and trace amounts of histidine, adenine, uracil, lysine, and leucine (Gutz *et al.* 1974). The peptone in YPD induces lysis of *S. pombe ura4*^−^ mutants (Matsuo *et al.* 2013) and creates a stress in all strains such that they are induced to mate while growing in what should be a rich medium. Thus, YPD does not provide a permissive growth condition for *S. pombe*. In addition, the standard defined medium used for *S. cerevisiae*, SC or SD medium, is inferior for *S. pombe* growth to Edinburgh Minimal Medium (EMM) (Gutz *et al.* 1974), although the basis of this growth difference is not known.

### Ploidy and meiosis

The difficulties of creating and maintaining stable diploid strains of *S. pombe* were mentioned earlier. *S. cerevisiae* cells mate in nutrient-rich conditions but require a strong starvation signal to induce meiosis. In contrast, *S. pombe* cells require starvation conditions to mate, but nearly all diploid zygotes sporulate immediately (Figure 3 and File S3). While this is advantageous for tetrad dissection and strain construction, it poses a challenge for experiments that require diploid strains, such as dominance and complementation tests. Diploid strains are also used to introduce a change into the genome that may be lethal or deleterious in a haploid strain, such as a gene deletion. By altering one allele in a diploid, one can first confirm that the change has been introduced before sporulating the diploid to determine the consequence of the change in haploid strains.

Two methods exist for constructing and maintaining *S. pombe* diploids. First, the *ade6-M210* and *ade6-M216* alleles both confer adenine auxotrophy, but they complement intragenically such that the heterozygous diploid (*ade6-M210/ade6-M216*) grows in medium lacking adenine. Since these mutations are in the same gene, even when some diploid cells enter meiosis and produce spores, these haploid spores are virtually all adenine auxotrophs because of the tight linkage between the *ade6-M210* and *ade6-M216* alleles. This allows one to use Ade*^+^* selection to maintain the diploid strain in the absence of contaminating Ade^+^ haploid progeny. Second, strains expressing the *mat2-102* allele can mate with either an *h*^+^ or *h*^−^ heterothallic strain to generate a diploid. The diploid with *h*^+^ immediately enters meiosis in the normal way, and sporulation ensues. However, in the diploid with *h*^−^, neither haploid parent expresses a functional *mat-Pm* gene, which is required for meiosis, so the diploid nucleus persists, generating a stable diploid line. This allows one to select for diploid cells by using two haploid parents carrying complementary selectable markers (*e.g.*, two different auxotrophies). Because meiosis is completely suppressed, there is no possibility of haploid progeny arising by meiotic recombination that would share the same phenotype as the diploid. Spontaneous mitotic haploidization following chromosome loss can occur. This haploidization can be stimulated by exposure to a low concentration of the microtubule-destabilizing drug benomyl (Alfa *et al.* 1993). Since it occurs in the absence of meiotic recombination, this method can be used to map mutations to one of the three *S. pombe* chromosomes, with the caveat that a mitotic recombination event could produce recombinant chromosomes in rare haploid segregants. One can distinguish between the original diploid strain and the viable haploid derivatives by plating for colonies on medium containing the vital stain Phloxine B, which stains dead cells (Gutz *et al.* 1974; Tange and Niwa 1995). Diploid colonies contain many more dead cells than haploids, so they are dark red on Phloxine B–containing plates, while haploid colonies are light pink. Phloxine B is also be used to detect rare diploids that arise in a culture, presumably as a result of chromosomal nondisjunction. These diploids grow faster than the haploid parent and will overtake a culture if one does not initiate an experiment from a single colony. Such diploids are mating competent but produce mostly inviable aneuploid progeny when crossed with a haploid strain owing to the creation of a triploid zygote.

### Homologous recombination

One of the key strengths of both *S. pombe* and *S. cerevisiae* for molecular genetics studies is the ability to introduce DNA into the chromosome via homologous recombination during vegetative growth. This allows one to delete genes, construct translational fusions, or even construct specific mutant alleles. However, the relative ratio of homologous *vs.* nonhomologous insertion events is lower for *S. pombe* than for *S. cerevisiae*. The relatively high proportion of nonhomologous events in *S. pombe*, in which a small linear DNA molecule integrates at a site in the genome other than at the location targeted by the sequences at the ends of the molecule, can create difficulties. While different laboratories take different approaches to increase the efficiency of targeted DNA insertions, it is generally sufficient to create a PCR product carrying the transforming DNA that has 60 bp of targeting sequences flanking the product (Bähler *et al.* 1998).

### RNAi

Unlike *S. cerevisiae*, *S. pombe* has retained through evolution the genes that encode the RNAi machinery, including the Dcr1 DICER ribonuclease, the Rdp1 RNA-dependent RNA polymerase, and the Ago1 Argonaute family member. Research on RNAi in *S. pombe* has not only contributed to our understanding of its mechanism and consequences but also helps to explain other biological differences between fission and budding yeasts. Loss of any RNAi genes leads to an increase in the presence of transcripts from the heterochromatic region of *S. pombe* centromeres as well as defects in chromosome segregation during mitosis (Volpe *et al.* 2002, 2003). Thus, the absence of RNAi in budding yeast may be linked to loss of large, repetitive centromeres as found in *S. pombe*, which, in turn, may explain the relatively small size of *S. cerevisiae* chromosomes that appear to segregate in mitosis by attachment to a single microtubule (Peterson and Ris 1976). RNAi also appears to play a modest role in regulating expression of Tf2 retrotransposons in *S. pombe* (Hansen *et al.* 2005) and the more abundant retrotransposons found in the related fission yeast *Schizosaccharomyces japonicus* (Rhind *et al.* 2011). Because RNAi regulates retrotransposon expression in higher organisms (Tabara *et al.* 1999), it has been suggested that the loss of RNAi in *S. cerevisiae* could have led to the increased expression of reverse transcriptase activity that ultimately led to the loss of introns in the genomic DNA (Aravind *et al.* 2000). This might explain why *S. pombe* is more like metazoan cells than *S. cerevisiae* with regard to both the presence of introns and the retention of RNAi.

### Cell cycle

*S. pombe* and *S. cerevisiae* have both contributed greatly to our understanding of events and processes that determine and regulate passage through the eukaryotic cell cycle. It is instructive to compare the cell cycles of the two yeasts to appreciate both their similarities and their differences. *S. cerevisiae* cells start their cycles in G_1_ as unbudded cells with unreplicated (1c) DNA content. Prior to “start,” a point in G_1_, cells are uncommitted to the mitotic cycle or other developmental pathways (Hartwell *et al.* 1974), while completion of “start” commits the cell to the ongoing mitotic cycle. The G_1_ phase lasts about one-quarter of the cell cycle and is followed by the S phase, a further third of the cycle. There is arguably no true G_2_ phase because formation of the mitotic spindle starts during late G_1_ or early S phase and does not depend on completion of S phase. Thus, the spindle persists through most of the cell cycle, though its final elongation, which separates the chromosomes, takes place well after the end of S phase. Following mitosis, the (larger) mother and (smaller) daughter cells separate, the mother embarking on a new cycle and the daughter becoming a mother cell, giving rise to a new bud. In contrast, *S. pombe* cells complete their short G_1_ and S phases very soon after mitosis so that by the time of cell division, virtually every cell is already in G_2_, and this phase comprises about three-quarters of the cycle (Nasmyth *et al.* 1979). The mitotic spindle is present for only a short period, as in most eukaryotes, and a symmetric cell division ensues. Other genetic (Nurse 1975) and physiologic studies (Fantes and Nurse 1977, 1978) indicated the presence of a major regulatory point between G_2_ and M controlling entry into mitosis.

Initial studies of the cell cycles of the two yeasts did not reveal any similarities in the underlying control mechanisms. Indeed, for a considerable time it was believed that the budding yeast cell cycle was controlled only in G_1_, while the fission yeast cell cycle was regulated at the G_2_/M boundary. Therefore, it was surprising when *S. pombe* was shown to have a G_1_ control point despite the shortness of this phase of the cell cycle (Nurse and Bissett 1981). Furthermore, the *S. pombe* G_1_ and G_2_ control points turned out to share a key component, the Cdc2 gene product, later shown to be a protein kinase (Simanis and Nurse 1986). The *S. cerevisiae* homolog of Cdc2, Cdc28, was known to be required for G_1_ progression and was subsequently determined also to be needed at later stages of the cycle. Cdc2 and Cdc28 were shown to be functional homologs in that the *CDC28* gene could rescue the defect of a *cdc2* mutant and vice versa (Beach *et al.* 1982). Given the major differences between the cell cycles of the two yeasts and the large evolutionary distance between them, Nurse argued that if two disparate yeasts shared the Cdc2/28 function, other eukaryotes such as humans also might do so. This idea was proven by the isolation of a human Cdc2 homolog based on its ability to rescue the cell cycle defect of a *S. pombe cdc2* mutant (Lee and Nurse 1987). This discovery was the basis of the awarding of the Nobel Prize to Nurse a decade later, shared with Lee Hartwell for his pioneering studies of cell cycle genes in *S. cerevisiae* and with Tim Hunt for his identification of the cyclin partner to Cdc2.

### Coping with DNA damage during the cell cycle

Cells face intermittent threats to their survival and fitness. One of these is damage to nuclear DNA, which can arise spontaneously or following exposure to one of several environmental agents. Particularly damaging is ionizing radiation, which can cause double-strand DNA breaks. If left unrepaired, these breaks can lead to loss of large chromosomal regions and cell death. A very effective way to repair a broken chromosome is to copy the information from the corresponding sequence on another DNA molecule. This works well for diploid organisms because there are always at least two copies of a given chromosomal region present, but a haploid cell in G_1_ phase has only a single copy of most sequences. Two quite different strategies have evolved for dealing with double-strand breaks in the two yeasts, and they relate to the different lifestyles of the yeasts.

A pair of haploid *S. cerevisiae* cells will mate with each other independently of the nutritional environment, provided that they are of opposite mating type, to form a mitotically stable diploid cell that proliferates indefinitely, as long as nutrients are available. Following starvation, the diploid undergoes a meiotic division to generate four ascospores, two of each mating type. On return to growth medium, the ascospores germinate and, unless artificially separated, mate with one another, once again generating diploids. Thus, although laboratory *S. cerevisiae* strains can be maintained as haploids, this is not the natural state for the organism. The diploid state ensures that the cell always has two copies of each genomic sequence and can deal effectively with broken chromosomes by copying the intact version of the damaged sequence from the chromosomal homolog. Thus, *S. cerevisiae* cells can have a long G_1_ phase and still survive an otherwise lethal DNA-damaging event.

However, *S. pombe* cells will not mate with one another unless starved. Following starvation for (usually) nitrogen, cells of opposite mating types form a zygote that generally enters meiosis directly, with no extended diploid phase. The four resulting haploid ascospores each will proliferate as a haploid clone. Thus, the default lifestyle of *S. pombe* is as a haploid; diploid strains are unstable and difficult to propagate in the laboratory. So how do haploid *S. pombe* cells survive a double-strand DNA break, given that they have no sister chromosome to copy? The answer is that while the cells only have a single copy of each chromosome, the cells replicate their DNA and enter G_2_ very early in the cell cycle so that the chromosomes are composed of two sister chromatids. Breakage of one chromatid therefore can be repaired using the intact chromatid as a template. This shows how these yeasts achieve the same goal via two distinct mechanisms.

## Genetic Toolkit: As Illustrated by a Plasmid-Based Mutant Screen

Yeast molecular genetics projects commonly follow one of two strategies. The first involves the isolation of strains possessing chromosomal mutations that confer a desired or distinct phenotype (we sometimes find things we were not looking for but cannot ignore), followed by identification of the gene or genes of interest. An excellent description of this approach has been provided in the Primer on the use of *S. cerevisiae* (Duina *et al.* 2014) and can serve as a template for similar studies in *S. pombe*. The second strategy involves a plasmid-based screen. This could be a screen of genomic or complementary DNA (cDNA) libraries (Table 1) for plasmids that confer a desired phenotype or a screen to identify mutant alleles of a specific cloned gene of interest. This latter scenario serves as the focus for this Primer on *S. pombe* molecular genetics methods and tools. While our scenario will involve screening for mutations of a heterologously expressed gene in *S. pombe*, the same approach can be used to identify mutations of interest in an *S. pombe* gene carried on a plasmid.

**Table 1:** *S. pombe* genomic and cDNA libraries

Library	Insert	Marker	Reference
pWH5 (*Sau*3AI) library	Partial *Sau*3AI digest-genomic—10 kb	*LEU2*^+^	Feilotter *et al.* 1991
pWH5 (*Hin*dIII) library	Partial *Hin*dIII digest-genomic—10 kb	*LEU2*^+^	Feilotter *et al.* 1991
pBG2 library	Partial *Sau*3AI digest-genomic—2.9 kb	*his3*^+^	Ohi *et al.* 1996
pEA500 library	Partial *Sau*3AI digest-genomic—3.9 kb	*his7*^+^	Wang *et al.* 2005
pLEV3/SPLE1	*adh1*-driven cDNA (>1-kb transcripts)	*LEU2*^+^	Janoo *et al.* 2001
pLEV3/SPLE2	*adh1*-driven cDNA (<1.6-kb transcripts)	*LEU2*^+^	Janoo *et al.* 2001
pREP3X library	*nmt1*-driven cDNA	*LEU2*^+^	Edgar and Norbury, unpublished data

Four genomic and three cDNA libraries that have been used to clone *S. pombe* genes are listed. Information regarding the cloning vector used, the nature of the insert DNA, the selectable marker, and the promoter driving expression of the inserts for the cDNA libraries are given. For genomic libraries, the average size of the inserted DNA is provided. The SPLE1 and SPLE2 cDNA Libraries contain size-selected inserts.

When eminent British naturalist W. T. Pooh was about to search for something, “He thought he would begin the Hunt by looking for Piglet, and asking him what they were looking for before he looked for it” (Milne 1928). Similarly, in forward genetics screens, where the scientific question is “What genetic events confer the desired phenotype?” one must first determine what phenotype is being sought. This phenotype must vary as a function of the activity of the gene of interest. For genes expressed from a plasmid, the level of activity is controlled by plasmid copy number, the strength of the promoter used to drive expression, and the specific activity of the encoded protein. The phenotype also will depend on the host strain used for the screen. When searching for loss-of-function alleles, the level of expression of the target gene is generally not an issue, but when looking for gain-of-function alleles, the level of expression is important because overexpression of the wild-type allele may produce the desired mutant phenotype. In this situation, one must test different expression vectors to identify one that does not alter the relevant phenotype of the cell when expressing the wild-type protein.

**Figure 5:**
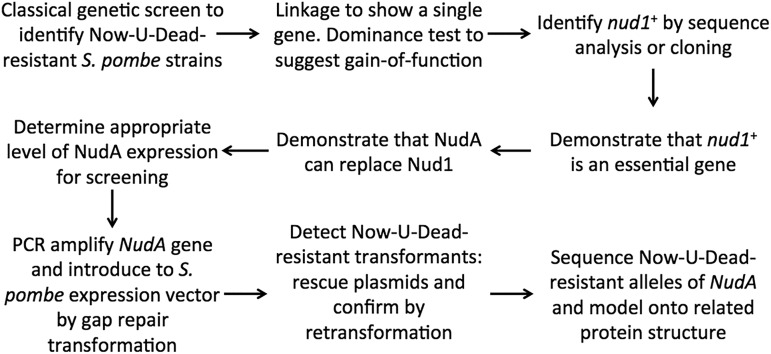
Outline for genetic analysis of *nudA* gene.

In our scenario, you may have discovered through a classical genetics approach (Figure 5) that the target of the drug Now-U-Dead is conserved between *S. pombe* and *Plasmodium falciparum*, the causative agent for malaria. These studies began by isolating *S. pombe nud1*^R^ mutants that are resistant to this drug, which is normally toxic to both *S. pombe* and *P. falciparum*. Further analyses showed that these mutant alleles are genetically linked to each other (all progeny from a cross of any two resistant strains are resistant to Now-U-Dead) (Figure 5) and are dominant to the wild-type allele that is present in strains that are sensitive to Now-U-Dead (*i.e.*, *nud1*^R^/*nud1*^+^ heterozygous diploids are resistant to Now-U-Dead). You also tried to isolate strains that are resistant to Now-U-Dead using one of two transposon-insertion strategies (Park *et al.* 2009; Li and Du 2014). These approaches generate large numbers of mutants for which the presence of the foreign transposon allows rapid identification of the insertion site. However, these approaches failed to produce resistant strains. These results suggest that either the gene or genes in question are essential or that a loss-of-function mutation does not confer resistance to Now-U-Dead.

You then identified the *nud1*^+^ gene by one of two methods (Figure 5). The choice of method will depend on resources available and likely will change with time as next-generation high-throughput sequencing becomes commonplace (Koboldt *et al.* 2013). First, high-throughput sequencing of two or more independently isolated *nud1*^R^ strains identified a single gene that is altered in all these strains relative to the 972 reference genome sequence or your parental strain. A plasmid carrying a candidate *nud1*^R^ allele confers Now-U-Dead resistance to a strain that is sensitive to Now-U-Dead. Second, candidate genes were identified from a cDNA library (Table 1 provides a list of genomic and cDNA libraries used to clone *S. pombe* genes) by their ability to confer resistance to Now-U-Dead when expressed at high levels. The corresponding alleles in *nud1*^+^ and *nud1*^R^ strains were sequenced to determine which gene identified in the screen is altered in *nud1*^R^ strains. Finally, after either method of gene identification, you confirm that expression of a candidate *nud1*^R^ allele, but not the *nud1*^+^ allele, in a *nud1*^+^ strain confers Now-U-Dead resistance.

You subsequently showed that *nud1*^+^ is an essential gene (Figure 5). This information is present in the PomBase database; although had the database suggested that *nud1*Δ cells are viable, you should still confirm this independently (Guo *et al.* 2013). This is done by deleting one copy of the gene in a diploid strain, inducing sporulation, dissecting tetrads, and determining that haploid progeny carrying the *nud1*Δ deletion are nonviable. The fact that *nud1*^+^ is essential suggests that Now-U-Dead kills *S. pombe* by inactivating or partially inactivating Nud1. You also showed that the *P. falciparum nudA* gene, whose sequence is homologous to *nud1*^+^, can replace *nud1*^+^ to keep a *nud1*Δ strain alive (Figure 5). (A *nud1*Δ/*nud1*^+^ heterozygous diploid strain with a plasmid that expresses *P. falciparum nudA* produced viable *nud1*Δ progeny that carry the plasmid.) To facilitate malaria research, you would like to identify mutations in the *P. falciparum nudA* gene that confer resistance to Now-U-Dead with the aim of developing other drugs targeting NudA that remain effective against the mutant NudA protein. In this way, drug resistance would be much less likely to arise in patients who receive a combination of drugs that target different sites in NudA.

Since *S. pombe* Nud1 is believed to be inactivated by Now-U-Dead, the host strain can be *nud1*^+^, and you can screen for Now-U-Dead resistance among transformants that carry an autonomously replicating plasmid expressing the *P. falciparum nudA* gene. Many *S. pombe* plasmids use the *S. cerevisiae LEU2*^+^ gene, which complements a mutation in the *S. pombe leu1* gene (Beach and Nurse 1981), or *S. pombe* biosynthetic pathway genes such as *ura4*^+^, *his7*^+^, *his3*^+^, and *lys2*^+^ (Bach 1987; Apolinario *et al.* 1993; Burke and Gould 1994; Hoffman and Hoffman 2006). In contrast, drug selection markers, which do not require host strains to possess auxotrophic mutations, are used more often as templates to generate PCR products for integration into the *S. pombe* genome to delete or tag target genes (Bähler *et al.* 1998; Tasto *et al.* 2001; Sato *et al.* 2005). Unlike in *S. cerevisiae*, centromeric plasmids are not used in *S. pombe* because *S. pombe* centromeres are too large to incorporate into traditional cloning vectors (Clarke *et al.* 1986). Therefore, *S. pombe* plasmids are present in multiple copies per cell, which contributes to the level of expression of the cloned gene, as does the strength of the promoter used to drive expression (Table 2).

**Table 2:** Promoters used in *S. pombe* research

Promoter	Strength	Regulation	Other comments	Reference
*nmt1*	Strong	Regulated*^a^*	Moderate expression even when repressed.	Forsburg 1993; Maundrell 1993
*nmt81*	Moderate	Regulated*^a^*	Weak expression even when repressed.	Forsburg 1993; Maundrell 1993
*nmt41*	Weak	Regulated*^a^*		Forsburg 1993; Maundrell 1993
*urg1*	Strong	Regulated*^b^*	Not regulated when on a plasmid.	Watson *et al.* 2011, 2013
*adh1*	Strong	Constitutive		Russell and Hall 1983; McLeod *et al.* 1987; Forsburg 1993
*tif471*	Moderate	Constitutive		De Medeiros *et al.* 2015
*lys7*	Weak	Constitutive		De Medeiros *et al.* 2015

a *nmt*-based promoters are repressed by thiamine (vitamin B_1_), but induction is relatively slow.

b *urg1* promoter is rapidly induced by exogenous uracil.

**Figure 6:**
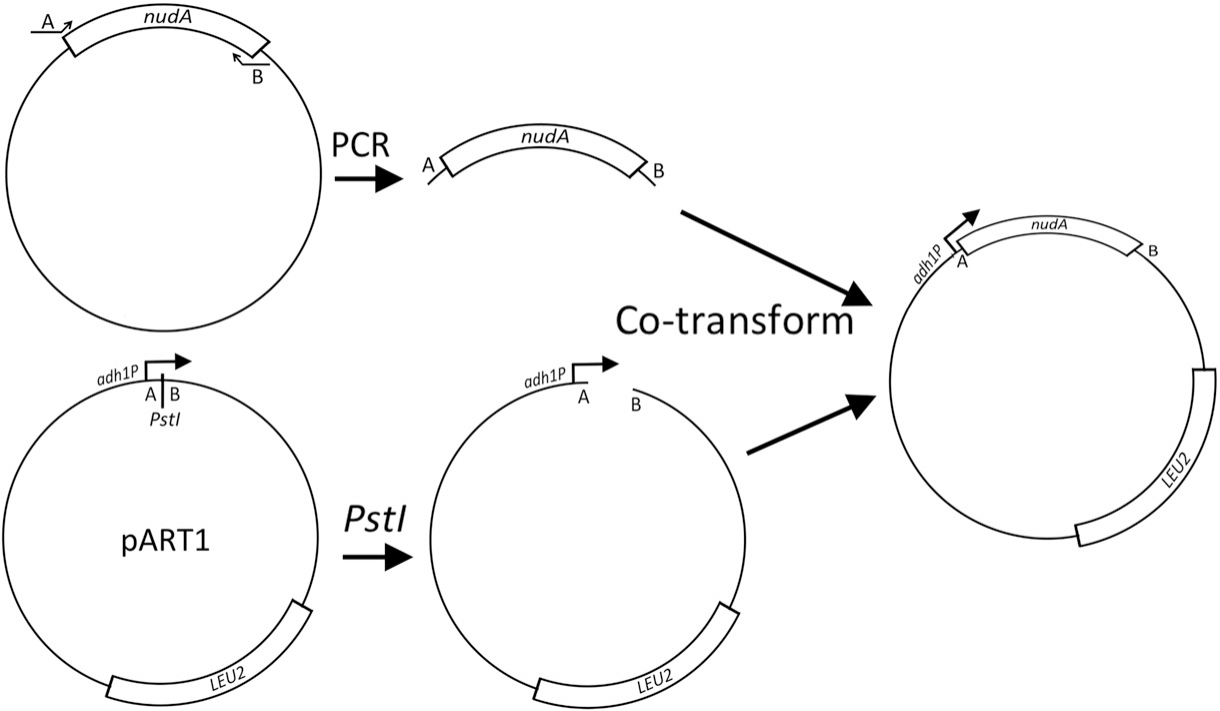
Gap-repair cloning of the *nudA* gene. The *nudA* gene is amplified by PCR using oligonucleotides that target its insertion into the linearized expression vector pART1 by gap repair transformation. This places the *nudA* open reading frame under the control of the *S. pombe adh1* promoter to drive its expression in fission yeast.

To clone the *P. falciparum nudA* gene into an *S. pombe* expression vector, use PCR to amplify *nudA* from a cDNA clone or library with primers whose 5′ ends contain 45–60 bp of sequence found on either side of a unique restriction site that is downstream of a promoter in a replicative *S. pombe* expression vector such as pART1 (McLeod *et al.* 1987) or pREP1 (Maundrell 1993) (Figure 6). To promote homologous recombination, 45 bp of targeting sequence is sufficient for integrating DNA into plasmids, while 60 bp is safer for chromosomal insertions. The 3′ ends of these primers anneal to the ends of the *nudA* open reading frame, so the final product is the *nudA* gene flanked by sequences that target the insertion of this DNA into the cloning vector. Cotransforming an *S. pombe* strain with the PCR product and the linearized expression vector produces plasmids with the PCR product inserted into the vector by gap repair (Kostrub *et al.* 1998). These transformants form colonies on the defined medium EMM lacking leucine as a result of expression of the *LEU2*^+^ selectable marker. However, *adh1* and *nmt1* (in the presence or absence of thiamine) express NudA at such high levels that even transformants expressing the wild-type allele display some resistance to Now-U-Dead. In contrast, transformants expressing NudA from the moderate-strength *nmt41* promoter in pREP41, when grown in the presence of thiamine, remain sensitive to Now-U-Dead. This identifies a level of NudA expression that will facilitate the isolation and characterization of alleles of *nudA* that are resistant to Now-U-Dead (Figure 5).

Next, generate PCR products containing a low number of random mutations, including ones that produce drug-resistant proteins. PCR using the FailSafe PCR System (Epicentre Biotechnologies) produces both transition (purine-to-purine) and transversion (purine-to-pyrimidine) mutations to generate a greater diversity of amino acid substitution mutations than transition mutations alone (Ivey *et al.* 2010). This PCR reaction is carried out using *nudA* cDNA template and oligonucleotides that will target insertion into a linearized pREP41 vector, and the product is cotransformed with the linearized pREP41 vector. Once Leu^+^ transformants are visible on the plate (generally after 4–6 days of growth at 30°), replica plate them to medium containing Now-U-Dead to identify drug-resistant transformants. It may be useful to include the vital stain Phloxine B in the growth medium in case the cells in compound-sensitive colonies undergo a few rounds of division before dying, which may mask the fact that the cells have died.

Since Now-U-Dead resistance could arise from a chromosomal mutation in the *S. pombe nud1*^+^ gene, you must confirm that compound resistance is plasmid conferred. This can be done by rescuing the plasmids from yeast transformants into *E. coli*, isolating the plasmids, and retransforming the original host strain (all transformants should now be compound resistant). You can also test whether loss of the plasmid in the yeast transformant restores sensitivity to Now-U-Dead by growing transformants on rich medium [yeast extract medium with supplements (YES)] to allow for mitotic loss of plasmids and streaking for single colonies on YES medium to produce colonies that either lack or retain plasmids. These colonies are then replica plated to YES medium, EMM lacking leucine (to identify Leu^−^ colonies that have lost plasmid), and medium containing Now-U-Dead (to determine whether the Leu^−^ colonies have regained compound sensitivity). Once plasmids have been shown to confer compound resistance, you can sequence the mutant alleles to determine what residue or residues have been altered in the mutant sequence. If alleles carry multiple mutations, you may need to perform site-directed mutagenesis to create single amino acid substitution alleles and test their ability to confer drug resistance. This can be done using the CRISPR/Cas9 system, which has been used recently to directly edit *S. pombe* genomic sequences (Jacobs *et al.* 2014).

While there is not a crystal structure for the NudA protein, you may be able to use the program SWISS-MODEL to predict its tertiary structure and to identify the location of the altered residues (Schwede *et al.* 2003). In this way, you can identify a likely binding site for the compound that may provide guidance for the design of other compounds that target NudA and thus remain effective against forms of NudA that are resistant to Now-U-Dead. Additionally, if there is more than one compound that targets NudA, you can screen for resistant alleles to the individual compounds. These alleles then can be used to profile resistance to the various compounds to develop a drug combination to treat malaria patients so as to prevent the selection for drug-resistant strains of *P. falciparum*.

In addition to using *S. pombe* to detect alleles of *P. falciparum nudA* that are resistant to Now-U-Dead, you may study the function of the *S. pombe* Nud1 protein directly. Since *nud1*^+^ is essential, you could conduct a plasmid-based screen to identify temperature-sensitive *nud1* alleles to investigate what happens as cells lose Nud1 function. However, Now-U-Dead can be used for this purpose without the need for temperature-sensitive alleles. Instead, you will look for proteins that physically interact with Nud1. While there are many ways to do this, we will consider just two of them. First, you could take a two-hybrid screening approach (Chien *et al.* 1991) to look for *S. pombe* proteins that directly bind Nud1. To do this, you construct a translational fusion between the *nud1*^+^ open reading frame and the *S. cerevisiae* Gal4 DNA-binding domain in a plasmid such as pAS1 that can replicate in either *E. coli* or *S. cerevisiae* (Durfee *et al.* 1993). You then coexpress this “bait” plasmid, which carries a *TRP1*^+^ selectable marker, together with a library of *LEU2*^+^-marked “prey” plasmids (the insert DNA is randomly sheared *S. pombe* cDNA to create a collection of translational fusions to the Gal4 transcriptional activation domain) in an *S. cerevisiae* strain such as YRG-2 (Durfee *et al.* 1993). If the bait and prey proteins interact in a transformant, they form a protein complex that stimulates transcription of reporter genes.

Alternatively, you could look for proteins that form a complex with Nud1, via direct or indirect interactions, by constructing a tandem affinity protein (TAP)–tagged version of Nud1 using cassettes that tag either the N- or C-terminus of the protein (Tasto *et al.* 2001) at the *nud1*^+^ locus in *S. pombe*. The tagged Nud1 protein then can be isolated together with its binding partners by applying sequentially two different affinity purification methods, leading to a highly purified protein complex. You can determine the individual binding partners or detect all copurified proteins via mass spectrometry (Tasto *et al.* 2001). Either approach helps to identify the role of Nud1 by revealing binding partners that may have been studied by other laboratories.

## Using PomBase as a Research Tool

The rapidly increasing information available regarding individual gene products and the growing trend toward functional dissection of large numbers of genes in a single study have made the fission yeast MOD PomBase (http://www.pombase.org/) an increasingly important tool for data mining to generate hypotheses, design experiments, and analyze data (Wood *et al.* 2012; Mcdowall *et al.* 2014). The main tools provided by PomBase are summarized in Table 3.

**Table 3:** PomBase tools and resources

Tool/Resource	Purpose
Advanced search Query history	Find all gene products with a specific feature (*e.g.*, otology term, intron number, domain). Combine the output of searches (add, intersect, subtract).
Gene list search	Upload a user-defined list to compare with other lists.
GO slim	Access lists of genes annotated to “broad” biological processes.
Motif search	Identify lists of proteins matching user-defined amino acid sequence motifs.
Ensembl browser	Access sequence features in the context of the genome (*e.g.*, polyadenylation sites, transcriptome data, nucleosome position).
Comparative genomics	Synteny views (*S. octosporus*, *S. japonicas* genomes).
Compara	Search for orthologs/paralogs in Fungi, or in a pan-taxonomic comparison (eukaryotes), using Compara in the Ensembl browser.

PomBase is structured around gene pages that provide comprehensively curated data for each gene product. Here we describe the use of PomBase to find tentative clues for the cellular role of the elusive Nud1, identified in your studies. Although a minimal amount of curated data is available for previously unstudied genes such as *nud1^+^*, it is possible to gain some insights into the function of Nud1 from the GO, phenotype, protein domain, expression, and interaction data presented on PomBase.

The GO controlled vocabulary is used to consistently describe the gene product attributes of molecular functions, biological processes, and cellular component (localization or complex) (Ashburner *et al.* 2000). Because neither Nud1 nor its orthologs have been studied previously, no molecular function or biological process data are available. However, a genome-wide localization study using a GFP library provides cellular component (localization) data for ∼85% of fission yeast protein-coding gene products (Matsuyama *et al.* 2006). You see that Nud1 is found in the mitochondria (along with 757 other gene products).

The gene page also provides phenotype data using the Fission Yeast Phenotype Ontology (FYPO) together with supporting information describing alleles and experimental conditions (Harris *et al.* 2013). Analysis of a genome-wide deletion data set provides null mutant viability for >90% of protein-coding genes (Kim *et al.* 2010). In addition, a visual screen of this deletion resource for mutants affecting cell cycle (detected by effects on cell length) and cell shape has provided broad morphology phenotype annotations for many gene products (representing both viable cells and the terminal phenotypes of nonviable deletions) (Hayles *et al.* 2013). You see that deletion of *nud1*^+^ produces the phenotype “nonviable tapered cell,” similar to that of 134 other gene deletions.

Because mutations in functionally related genes often confer the same phenotype, studying a list of the genes coannotated to your ontology term of interest may shed some light on the biological role of Nud1. It could be even more informative to survey the list of genes that share these two annotations (cellular component “mitochondrion” and phenotype “nonviable tapered cell”). PomBase provides a tool (http://www.pombase.org/spombe/query/builder) that allows curated lists to be rapidly intersected, added to, or subtracted from. The intersect between cellular component “mitochondrion” and phenotype “nonviable tapered cell” shows that 95% of the genes with phenotype “nonviable tapered cell” encode mitochondrial proteins. In addition to GO terms and phenotypes, a wide range of other features can be queried, including intron number, protein length, modifications, protein families, and domains.

The protein motifs and domains predicted by the InterPro Consortium databases can be powerful functional indicators (Mitchell *et al.* 2015). Even if your protein of interest only contains a domain of unknown function (DUF), it may be a member of a superfamily with a known fold, which can indicate some broad classification such as a catalytic activity or transport function.

The “quantitative gene expression” data on the gene page provides transcript expression level data for all gene products. The protein level during vegetative growth or quiescence is also available for most proteins (Marguerat *et al.* 2012; Carpy *et al.* 2014). This expression level and the presence or absence at specific cell cycle or life cycle stages may give insight into possible roles of your gene of interest.

Physical and genetic interactions are curated in collaboration with BioGRID (Chatr-Aryamontri *et al.* 2013) and displayed on the PomBase gene pages, and they are also available via network diagrams using esyN (Bean *et al.* 2014). Physical interactions can link a gene product to processes based on the roles of interacting partners. There are no high-throughput physical interaction data sets for fission yeast at the time of this writing, so it is highly unlikely that there would be any physical interaction data for an unstudied gene. However, genome-wide genetic interaction data sets are becoming available (Ryan *et al.* 2012).

Other information presented on the gene pages includes protein features, protein modifications, human and *S. cerevisiae* orthologs, disease associations, and quantitative protein and RNA expression levels. A link from each gene page provides access to an Ensembl browser hosting sequence-based features including nucleosome positioning, polyadenylation sites, and transcriptomics data displayed in the context of the genome sequence.

## Using PomBase for Data Mining

Although the primary use of PomBase is to look up information on specific genes and their products, MODs are becoming increasingly pivotal during data analyses or for data mining to identify candidate genes of interest before embarking on any experiments. Ontologies provide a critical component of this data-mining capability because the ontology structure [reviewed in Washington and Lewis (2008)] enables powerful database querying. Increasingly, gene lists are generated as the output of a functional genomics experiment (*e.g.*, differentially expressed genes or the results of a phenotypic screen). You can import any user-defined gene list into the PomBase Query Builder and combine it with any of the available biological queries to evaluate the intersection of any feature with your list and to identify attributes that may be over-represented in your gene list relative to the whole genome.

One of the main uses of GO annotation is to analyze lists of genes or summarize experimental results by GO Enrichment analysis or GO slimming. GO Enrichment describes the identification of any statistically over-represented GO terms in a list of genes using one of the many GO Enrichment tools (*e.g.*, http://geneontology.org/ on the GO website). A GO slim is a set of broad GO terms used to provide a summary of the GO annotations for genes in a data set [*e.g.*, by the GO Term Mapper tool (http://go.princeton.edu/cgi-bin/GOTermMapper)]. GO slimming also can be used to summarize an organism’s total functional capabilities; a GO biological process slim classification for the entire fission yeast gene product set is on the PomBase website (www.pombase.org/browse-curation/fission-yeast-go-slim-terms).

## Notable Advances from Research on Fission Yeast

The yeasts *S. pombe* and *S. cerevisiae* were propelled to the forefront of research to characterize basic cellular mechanisms because they are (1) single-cell organisms, (2) eukaryotes, (3) able to be maintained as a haploid or diploid, (4) able to maintain autonomously replicating plasmids, and (5) organisms with highly active homologous recombination machinery that facilitates targeted changes to the genome. Research using *S. pombe* has made significant contributions to our understanding of biology in several key areas.

### Cell cycle

*S. pombe* research has played a unique and highly prominent role in understanding the cell cycle. Nurse combined Mitchison’s interest in the cell cycle with Leupold’s genetic approaches to identify genes that play essential roles in cell cycle progression (Nurse 1975; Nurse *et al.* 1976). The best known and most important outcome of this work was the cloning of the *cdc2*^+^ cyclin-dependent kinase gene (Beach *et al.* 1982) and the critical demonstration that the human homolog could supply this function to a *cdc2^ts^* temperature-sensitive mutant (Lee and Nurse 1987). The mutant screens that identified *cdc2^+^* also led to the discovery of several genes whose products interact with or act on the Cdc2 kinase, such as Cdc25 [the phosphatase that activates Cdc2 by dephosphorylation of a crucial phosphotyrosine residue (Russell and Nurse 1986)] and Cdc13 (Nurse *et al.* 1976; Fisher and Nurse 1996). A separate study led to the isolation of mutants with reduced cell size, nearly all of which had a defective *wee1* gene, later shown to encode the tyrosine kinase that inhibits Cdc2 activity until cells are ready to pass from G_2_ to M phase (Nurse 1975; Nurse and Thuriaux 1980; Russell and Nurse 1986, 1987; Gould and Nurse 1989). Furthermore, the importance of cell size in cell cycle control was supported by the direct demonstration that cell size at division is homeostatically controlled. A cell that is larger or smaller than normal at birth will undergo division at close to the normal size, so nearly all the deviation from normal is corrected within the same cycle (Fantes 1977).

Strains carrying mutations in other *cdc* genes facilitated studies of important processes in addition to entry to mitosis: because they displayed defects in DNA replication, septum formation/septation, and cytokinesis. For example, Cdc1, Cdc6/Pol3, and Cdc27 are subunits of DNA polymerase delta (MacNeill *et al.* 1996), while Cdc18, Mcm2 (Cdc19), Mcm4 (Cdc21), Cdc23, and Cdc24 all play roles in either regulation of S phase or DNA replication itself (Coxon *et al.* 1992; Kelly *et al.* 1993; Forsburg and Nurse 1994; Aves *et al.* 1998; Gould *et al.* 1998). Cdc7, Cdc11, Cdc14, and Cdc16 are required for function of the septation initiation network (SIN) that monitors the completion of mitosis to regulate the start of cytokinesis (McCollum and Gould 2001). Finally, Cdc3 (profilin), Cdc8 (tropomyosin), Cdc12 (formin), and Cdc15 are all required for cytokinesis (Fankhauser *et al.* 1993; Balasubramanian *et al.* 1994; Chang *et al.* 1997). Thus, the study of mutations that cause cell cycle defects in *S. pombe* formed the basis of many distinct areas of research.

In addition to requiring dedicated gene products, progress through the cell cycle is influenced by both intrinsic and extrinsic conditions. There exist several cell cycle checkpoints at which the cell determines whether or not conditions have been met to allow progression to the next phase of the cell cycle. Several related DNA checkpoints act at various cell cycle stages (Carr 2004), while the spindle checkpoint acts to regulate exit from mitosis (Hauf 2013). Similar checkpoints exist in all eukaryotes, and many of the components are conserved.

DNA checkpoints detect damaged or unreplicated DNA and block cell cycle progress until the damage is repaired or replication is completed, when progress resumes. In *S. pombe*, the best understood is the G_2_/M checkpoint that prevents entry into mitosis while damaged DNA is present. Mutants defective in this checkpoint function have been isolated in both budding and fission yeast by their sensitivity to DNA-damaging agents such as radiation or agents that block replication such as hydroxyurea (Nasim and Smith 1975; Enoch *et al.* 1992). In outline, the checkpoint system works as follows: sites of damaged DNA are recognized by binding of checkpoint protein complexes, which activates a cascade of signaling components, ultimately preventing entry into mitosis. Gene products involved in binding to damaged or unreplicated DNA include the Rad3 protein kinase (Bentley *et al.* 1996) and its accessory factor Rad26 (Edwards *et al.* 1999). Further complexes involved in binding to sites of damage contain Rad17 (Griffiths *et al.* 1995), Hus1 (Kostrub *et al.* 1997), Rad1 (Sunnerhagen *et al.* 1990), Rad9 (Murray *et al.* 1991), and other proteins. Rad3 then interacts with other components, including the effector kinase Chk1 (Walworth *et al.* 1993; Al-Khodairy *et al.* 1994), whose activation results in increased tyrosine phosphorylation of Cdc2, which prevents entry into mitosis. The increase in Cdc2 phosphorylation appears to involve both downregulation of the Cdc25 phosphatase and upregulation of Wee1 (Furnari *et al.* 1997; Boddy *et al.* 1998; Raleigh and O’Connell 2000).

DNA checkpoints that act at other cycle stages—*e.g.*, to check that the DNA is properly replicated in S phase—share many of the components and complexes involved in the G_2_/M checkpoint, but there are also elements unique to one or other system. For instance, the effector kinase in the S-phase checkpoint is Cds1 (Murakami and Okayama 1995; Raleigh and O’Connell 2000) rather than Chk1 (Furnari *et al.* 1997; Boddy *et al.* 1998). Many of these components and mechanisms are conserved in mammalian cells, along with the functions of many more proteins involved in the detection and responses to DNA damage or stalled DNA replication (Sancar *et al.* 2004).

A significant difference between fission and budding yeast is that the final target of the G_2_/M checkpoint in budding yeast is not Cdc28 (the functional homolog of Cdc2) but Pds1, a protein whose destruction is required for exit from metaphase and entry into anaphase (Sanchez *et al.* 1999). The controls in mammalian cells are in this respect similar to those in *S. pombe*. The G_2_/M checkpoint is more complex than in *S. pombe*, but a major part of the G_2_ delay following DNA damage is due to a reduction in Cdc25 activity. This leads to an increased level of Cdc2/Cdk1 tyrosine phosphoryation on the equivalent residue to that of *S. pombe* Cdc2 and hence its inactivation. In response to DNA damage in G_1_, Cdc25A is phosphorylated and degraded so that it is unable to activate Cdk2–cyclin E, the cyclin complex required to initiate S phase (Sancar *et al.* 2004).

The observation that the nutritional composition of the growth medium influences cell cycle progress is an old one (Fantes and Nurse 1977), but until recently, the mechanism has not been clear. The fortuitous isolation of a mutant in which cell size was greatly affected by nutritional status (Ogden and Fantes 1986) led to the discovery and elucidation of the Sty1/Spc1 stress-activated MAP kinase signaling pathway that affects the rate of passage through the G_2_/M control. This nutritional control involves signaling through the TOR system and the Polo-like kinase Plo1, which acts indirectly to regulate the tyrosine phosphorylation status and hence activity of Cdc2 (Petersen and Nurse 2007; Hartmuth and Petersen 2009). In addition to influencing progress at the G_2_/M boundary, the Sty1/Spc1 pathway transduces nitrogen and glucose starvation stress as well as osmotic, oxidative, and heat stress to generate a spectrum of cellular responses (Chen *et al.* 2003).

### Chromosome dynamics

*S. pombe* also has been a key model for the study of biological mechanisms in the area of chromosome dynamics. Possibly owing to the large sizes of the *S. pombe* chromosomes, mechanisms associated with chromosome replication, recombination, and segregation during meiosis and mitosis are areas in which *S. pombe* research has played a central role. Mitsuhiro Yanagida and coworkers identified proteins required for proper chromosomal pairing and segregation in mitosis by isolating *cut* mutants that formed a septum prior to chromosome segregation (Hirano *et al.* 1986; Samejima *et al.* 1993). Yanagida’s contributions to this field have been recognized with several awards, including the Imperial Prize and Japan Academy Prize in 2003 and the Order of Culture Prize (Japan) in 2011. He was elected as a foreign member of the Royal Society (United Kingdom) in 2000 and a foreign associate of the National Academy of Sciences (United States) in 2011.

Because *S. pombe* centromeres are large, repetitive structures, similar to those of mammalian cells and unlike *S. cerevisiae* centromeres, research on chromatid cohesion at the centromeres (Watanabe and Nurse 1999; Kitajima *et al.* 2004), kinetochores and their interactions with the mitotic spindle (Goshima *et al.* 1999), and centromere clustering and movement during mitosis (Funabiki *et al.* 1993) have been central to developing our understanding of these processes in metazoan chromosomes. For example, two *S. pombe* Shugoshin homologs, Sgo1 and Sgo2, connect the spindle checkpoint proteins to sister chromatid cohesion (Kitajima *et al.* 2004). This work has prompted studies of vertebrate Shugoshin-like proteins to show that they are involved in the same interactions as in *S. pombe* (Salic *et al.* 2004; Kitajima *et al.* 2005) and in chromosomal instability associated with various forms of cancer (Kahyo *et al.* 2011; Yamada *et al.* 2012).

Similarly, studies of the structure and behavior of telomeres at the ends of these large chromosomes have led to important discoveries about teleomeric proteins (Cooper *et al.* 1997; Baumann and Cech 2001; Chikashige and Hiraoka 2001; Martin-Castellanos *et al.* 2005) involved in controlling the clustering and positioning of telomeres during meiosis that contribute to proper chromosome segregation as well as the pairing of chromosomes for homologous recombination.

In addition, *S. pombe* continues to be a key model organism for the study of DNA replication, with many of the original *cdc* genes being involved in various aspects of DNA replication. Current studies are examining the locations of origins of replication, the timing of origin firing, the regulation of replication fork movement, and the response to events such as replication fork stalling. *S. pombe* origins of replication are somewhat like those of higher eukaryotes in their lack of a conserved sequence element (Mojardin *et al.* 2013), thus making *S. cerevisiae* an outlier in this regard. Along with studies of DNA replication mechanisms, it has been shown that gene expression is affected by whether a DNA replication fork passes through the gene in the sense or antisense direction and that this is controlled by the location of surrounding origins, the timing of origin firing, and the presence of binding sites for the Sap1 or Reb1 proteins that block replication fork progression (Sanchez-Gorostiaga *et al.* 2004; Krings and Bastia 2005; Mejia-Ramirez *et al.* 2005). Replication fork arrest is also used to control the direction of replication through a site in the mating locus (Figure 4) that carries an imprint that controls which daughter cell will undergo a mating switch event (Dalgaard and Klar 2001). Further studies of replication origins, DNA replication, and the interplay between DNA replication and mating-type switching or gene expression in *S. pombe* undoubtedly will lead to new insights that will inform our understanding of mammalian molecular mechanisms.

### Chromatin, epigenetics, and gene expression

Both *S. pombe* and *S. cerevisiae* have been important organisms for the study of epigenetic control of gene expression. Expression of genes present at the *mat2-P* and *mat3-M* mating-type loci (Figure 4) and in telomeric and subtelomeric regions is silenced by a variety of mechanisms that ultimately affect the chromatin in these regions (Egel *et al.* 1984; Thon and Klar 1992; Thon *et al.* 1994; Allshire *et al.* 1995; Ekwall *et al.* 1996). In addition, the large heterochromatic centromeres of *S. pombe* have provided researchers with a third region in which to study silencing mechanisms (Allshire *et al.* 1994, 1995). Studies of heterochromatin formation at *S. pombe* centromeres have helped to establish a “histone code” that associates specific posttranslational modifications of histones with the transcriptional state of the associated chromatin (Jenuwein and Allis 2001). In *S. pombe*, Clr4 methylation of lysine 9 of histone H3 leads to the recruitment of the chromodomain protein Swi6 to nucleosomes containing the H3K9 mark (Nakayama *et al.* 2001). The establishment of transcriptionally silent heterochromatin also requires the function of the RNAi RITS complex (Volpe *et al.* 2002; Verdel *et al.* 2004), and Clr4, Swi6, and components of the RITS complex have been found to colocalize throughout the genome at heterochromatic regions (Cam *et al.* 2005). For an in-depth review of the many studies into the mechanisms and consequences of chromatin remodeling at *S. pombe* centromeres, see Allshire and Ekwall (2015). Finally, ncRNAs can affect chromatin remodeling and gene expression in an RNAi-independent manner. For example, transcriptional activation of the glucose-repressed *fbp1*^+^ gene involves the transient and stepwise expression of three ncRNAs that facilitate chromatin remodeling in the promoter region and the eventual expression of the mRNA (Hirota *et al.* 2008). Given the complexity of chromatin remodeling mechanisms at various regions of the *S. pombe* genome and the importance of this to both regulated gene expression and the maintenance of heterochromatic regions of the genome, this will remain a vibrant area of research for years to come.

## Conclusions

Over the past 60 years, *S. pombe* has risen from relative obscurity as a research organism to one of the two major yeast model systems, together with *S. cerevisiae*. Early research focused on studies of sexual development and the cell cycle, but with the increasing number of scientists drawn to this organism, there has been a significant expansion of the research areas in which *S. pombe* work has made substantial contributions. This trend undoubtedly will continue in the years to come. Along with the classical yeast genetics approaches described in the *S. cerevisiae* Primer (Duina *et al.* 2014) and here, the use of global expression studies (Mata *et al.* 2002; Chen *et al.* 2003; Marguerat *et al.* 2012) and screens of the *S. pombe* deletion collection (Kim *et al.* 2010) creates large data sets that are accessible through MODs such as PomBase. This provides newcomers to fission yeast research with the ability to generate hypotheses regarding previously unstudied genes so that they can create research projects that will add to our understanding of the biology of *S. pombe*. Such new knowledge will, in turn, provide new insights into the biology of nonmodel organisms such as pathogens and ourselves.

## 
